# A Hybrid Clustering–Classification Approach for Predicting Strength and Analyzing Material Composition of Geopolymers

**DOI:** 10.3390/polym18080959

**Published:** 2026-04-14

**Authors:** Yıldıran Yılmaz, Talip Çakmak, İlker Ustabaş

**Affiliations:** 1 Department of Computer Engineering, Recep Tayyip Erdogan University, Fener, Rize TR53100, Türkiye; 2 Department of Civil Engineering, Recep Tayyip Erdogan University, Fener, Rize TR53100, Türkiye; talip.cakmak@erdogan.edu.tr (T.Ç.); ilker.ustabas@erdogan.edu.tr (İ.U.)

**Keywords:** geopolymer, compressive strength, EDS analysis, hybrid machine learning, K-means clustering, SHAP analysis

## Abstract

The development of geopolymers as sustainable alternative binders has been accelerated by the environmental requirement to reduce the carbon footprint of cement. However, predicting their key properties, such as compressive strength, from their complex chemical composition remains a significant challenge. Although mixture ratios prepared on a macro-scale are widely used for quality control purposes, they do not account for the chemical structure, despite this having a direct impact on the materials’ structural properties. Predicting fundamental properties such as compressive strength from complex chemical compositions remains a significant challenge due to the nonlinear relationships between the elemental components. This research paper introduces a tailored hybrid machine learning framework that combines K-means clustering with classification algorithms. The method uses energy-dispersive X-ray spectroscopy (EDS) data to classify geopolymer samples into their specific mixture numbers, which allows scientists to predict material properties through compositional analysis. A new dataset featuring the elemental compositions of Si, Al, Na, Ca, O, and C, as well as the critical ratios of Si/Al and Ca/Si, was analyzed. The initial step involved clustering the data to discover natural compositional clusters, which served as the basis for training and testing five different classifiers, which included Random Forest (RF), Artificial Neural Networks (ANN), LightGBM, Naive Bayes (NB), and Linear Discriminant Analysis (LDA). The consequences proved that the hybrid method worked with outstanding efficiency. RF achieved the highest performance results through its 98% accuracy, 96% recall, 94% precision, and 95% F1-score results when it classified samples according to their clustered groups. SHAP (SHapley Additive exPlanations) and permutation feature importance analyses both showed that Si/Al proportion functioned as the most crucial predictive variable, while oxygen (O) content and silicon (Si) content followed in importance. The K-means cluster labels produced high accuracy results because they demonstrated that compositional data had strong natural groups, which matched the target property. The system delivers an efficient method which enables fast and dependable geopolymer property forecasts through direct analysis of chemical composition with chemical composition analysis, thus delivering essential information to enhance mix design processes and boost sustainable building material production.

## 1. Introduction

Concrete serves as a substantial material in construction schemes because of its ability to adapt to various uses and its low cost and simple handling requirements [1,2]. The main ingredient of concrete, ordinary Portland cement (OPC), produces 7–9% of global CO_2_ emissions because of its energy-demanding production process, which creates clinker [3]. The high environmental impact of current practices has led to the search for eco-friendly solutions, with geopolymers showing potential as a viable option [4,5]. Geopolymers function as aluminosilicate materials which become solid through alkali activation instead of traditional cement hydration while utilizing industrial by-products such as fly ash and slag to achieve significant reductions in carbon emissions [6,7,8]. Geopolymers form by virtue of the reaction between alkaline solutions and binding materials with high levels of silicate and alumina [9]. The process starts with the dissolution of aluminosilicates in an alkaline solution [10]. In the production of geopolymer materials, selecting a suitable binder is crucial because it affects important properties such as reactivity, strength, durability, and workability [8]. Geopolymers are frequently produced using FA, GGBS, silica fume (SF), and other waste materials, as well as important activators, such as sodium hydroxide (SH) and sodium silicate (SS) [11,12]. Alkali activators play an important role in geopolymer production because they activate aluminosilicate precursors. Numerous studies have been conducted on the binders commonly used in geopolymer production. However, significant knowledge gaps remain regarding the effects of new binder types on important geopolymer properties, such as reaction kinetics, material properties, and rheological behavior [13]. Reactivity is one of the most important factors in developing materials activated with alkali activators. In geopolymer production, only the amorphous parts of the materials react; the crystalline parts behave like filler materials [14]. The binders’ high crystallinity indicates that they cannot be dissolved by alkaline activators and will remain inert within the geopolymer matrix [15]. For this reason, applying a single approach to the development of binders is difficult. A technique based upon the materials’ chemical and mineralogical ingredients and physical features is therefore needed [16,17]. The Si/Al ratio and other factors in geopolymer matrices are responsible for the variability observed in these matrices [18]. Therefore, analyzing the composition of materials plays a crucial role in various scientific and industrial applications, ranging from researching construction materials to controlling the quality of production. The ability to accurately predict material properties depends on elemental composition analysis, which serves as a fundamental element for both material assessment and industrial process optimization. The conventional analytical procedures require significant time and financial resources because they need extensive laboratory testing and specialized knowledge for large-scale assessments.

Machine learning (ML) methods now serve as a robust method for material characterization. The methods enable scientists to discover hidden material properties through their ability to classify composite structures and their connections to compositional data. The combination of clustering and classification methods shows great potential in material research because it enables researchers to discover inherent data groupings while using these patterns to forecast material characteristics. Hu et al. [19] used ML frameworks, including SVM, GBDT, ANN, and RF, to estimate the CS of FA-based geopolymers. Their research found that CS results were primarily determined by curing temperature and water content, while Si/Al ratio and alkali activator module also produced measurable effects. Zeng et al. [20] used different operational models, which included DT, RF, XGBoost, and MLP, to predict CS results and flexural strength of geopolymers made from coal gangue-based fly ash and GGBS. Researchers performed multiple orthogonal experiments by replacing GGBS and FA in coal gangue-based geopolymers at predetermined ratios that they adjusted to achieve specific Si/Al ratios. The experimental results demonstrated that both Si/Al ratios and water-to-binder ratios had a vital impact on the execution of the geopolymers. Han et al. [21] applied multiple machine learning algorithms, including ET, BA, XGB, SVR, DT, RF, and KNN, to forecast the absorption capability of geopolymer materials. They observed that curing time had the most significant effect on geopolymer absorption properties and that chemical components such as Na_2_O/SiO_2_, H_2_O/Na_2_O, and Na_2_O/Al_2_O_3_ also significantly affected absorption. Afzali et al. [22] analyzed the properties of metakaolin-based geopolymer concretes using machine clustering and classification algorithms with different working principles, such as ANN, SVM, DT, RF, and GBM. The study demonstrated that essential factors, which included the coarse-fine aggregate ratio, the H_2_O/Na_2_O molar proportion, and the additional water component, produced a major influence on the CS of MK-based geopolymer concrete. Bin et al. [23] employed their optimized ML frameworks to forecast the CS of their FA- and GGBS-based geopolymer composites. The team achieved their best performance results when they obtained an R^2^ value of 0.962 and an RMSE value of 5.6991. Stel’makh et al. [24] employed seven distinct ML models to foretell the CS of geopolymer concretes. The kNN model achieved the peak prediction execution, with R^2^ = 0.9996, RMSE = 0.63, and MAPE = 1.62. Yilmaz et al. [25] developed machine learning models with various operating principles, including LR, deep neural networks (DNN), RF, kNN, and XGBoost. These models were employed to foretell the FS and CS of geopolymer composites made from waste and natural materials, such as GW, FA, and OB. The XGBoost model produced the peak prediction execution with an R^2^ valuation of 0.981. The ranking of the other algorithms’ prediction performance was as follows: RF, DNN, kNN, and LR. Ngo et al. [26] created an ML framework, including GBRT, AdaBoost, XGBoost, and RF, to predict the CS and water resistance of geopolymer-stabilized compacted soil. The XGBoost model achieved the peak anticipation performance with an R^2^ valuation of 0.916. Another important performance metric, mean squared error (MSE), was 0.267, 0.536, 0.827, and 0.374 for the XGBoost, RF, AdaBoost, and GBRT algorithms, respectively. Cao et al. [27] used various ML methods to predict the CS of fly ash-based geopolymer concrete. The XGB model produced the most accurate predictions, with an R^2^ value of 0.98. This was followed by the SVM and MLP models, which produced R^2^ values of 0.91 and 0.88, respectively. Cakmak and Ustabas [28] applied tree-based models, including RF, ET, DT, and GBR, to foretell the CS of geopolymer mortars, comprising SF and obsidian. The researchers evaluated different machine learning systems by running tests from multiple training-test ratio setups. The study tested prediction performance through various training-testing split ratios, which included 0.6–0.4, 0.7–0.3, 0.8–0.2, and 0.9–0.1. The GBR algorithm produced its highest prediction results through an R^2^ value, which reached 0.972. The DT model achieved higher performance than both the ET and RF models. Harmaji et al. [29] used ML methods to predict the workability and CS of fly ash-based geopolymers, which contained both low and high calcium content. The XGBoost model produced R^2^ values of 0.96 and 0.89 for workability and CS predictions, respectively. Nazar et al. [30] examined the employability of ML frameworks, including ANN, ANFIS, and GEP, for estimating the slump and strength values of geopolymer materials containing FA. ANFIS showed the strongest strength prediction results, while ANN produced the most accurate slump prediction results among the different models. The different performance between the models occurs because they learned from different states of the dataset. Wang et al. [31] used multiple ML frameworks that included ANN, ANFIS, and GEP to predict CS of geopolymer concrete, which contains FA and GGBS. The GEP model delivered the highest results because of its R value, which reached 0.99, although all three algorithms showed strong predictive capabilities. The ANN framework followed with an R value of 0.97, and the ANFIS framework followed with an R valuation of 0.94. Shen et al. [32] developed machine learning models, including XGB, SVR, RF, GPR, GB, and CART, to estimate the autogenous shrinkage values of geopolymers containing slag and FA. GB and XGB models executed the peak prediction performance, with an R^2^ valuation of 0.95. The top performance of the models develops through this sequence XGB > GB > GPR > RF > SVR > CART. The studies present in this literature are shown in Table 1.

The reviewed literature demonstrates that machine learning methods are being used increasingly in material science to forecast the mechanical properties of geopolymers and cement-based materials. The three individual frameworks, RF, XGBoost, and ANNs, demonstrate strong predictive abilities, but their actual predictive ability depends on the characteristics of the dataset and the chosen modeling techniques. The existing conventional methods show successful results, but researchers have not yet explored hybrid methods that combine clustering with classification for predicting composition properties. The development of advanced hybrid systems will help to close this gap by delivering better predictive results, system stability, and model performance across different situations. The current research project aims to evolve a hybrid ML method, which improves the prediction accuracy of geopolymer material characteristics. The main objectives of this research study are:

1. The development of a hybrid model which unites K-means clustering and machine learning classification methods to analyze material composition.
2. The evaluation of multiple classification algorithms, which include RF, NB, ANNs, LightGBM, and LDA.
3. The presentation of results which demonstrate how clustering–classification techniques improve anticipation accuracy when compared to standard classification techniques.
4. This research delivers information about material composition data, which shows how those groups connect to material characteristics.

The system uses a hybrid model, which unites clustering and classification methods, because researchers believe that pre-clustering data enables better pattern detection, which improves subsequent classification accuracy. Our method starts with natural group detection for compositional data, which helps us to improve both predictive model accuracy and result understanding. The field has achieved significant progress, but researchers still investigate only a small set of research variables while failing to recognize the natural data groupings present in their datasets. Therefore, this study proposes a hybrid clustering–classification approach based on EDS data. The essential innovation of this study is the development of a holistic approach based on EDS analysis that goes beyond the influence of traditional, macro-scale mixture calculations on structural properties. This enables the model to operate independently of prior mixture design information, providing a high-resolution prediction tool that captures chemical heterogeneity, a factor that is often overlooked in the literature on traditional concrete technology.

## 2. Methodology

This study used a dataset of chemical components obtained from EDS analysis of geopolymer materials. This dataset includes input properties, such as silicon (Si), aluminum (Al), and calcium (Ca), as well as other important chemical components critical for geopolymer materials. The output parameter is CS. This dataset was created after several important stages, including calculating the geopolymer sample mixture using the necessary materials, casting, thermal curing, and performing mechanical and microstructural analyses.

**Figure 1:**
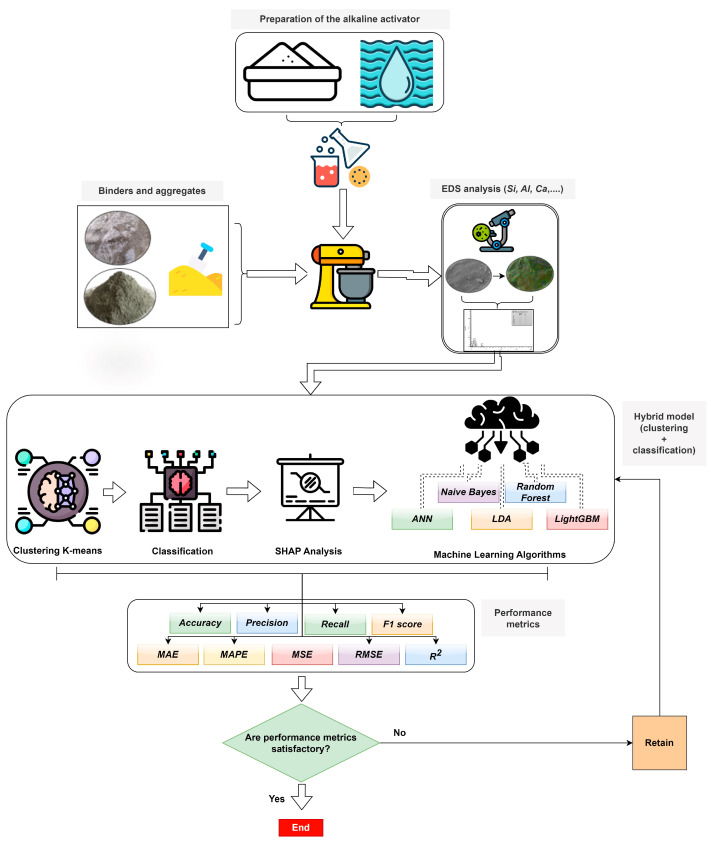
The main flow diagram, which represents the research methodology of the study.

Our dataset was used for clustering and classification purposes with classification and K-means clustering algorithms, such as RF, LDA, NB, LightGBM, and ANN. Different statistical performance measures were exercised to objectively appraise the execution of the classification and clustering methods and algorithms. Furthermore, cross-validation analyses were performed to augment the generalization performance of the frameworks, increasing their stability and reliability. Additionally, SHAP and permutation feature importance assay were conducted to ascertain the influence of input features on output parameters. Figure 1 provides a flowchart depicting all these processes.

### 2.1. Dataset Description and Preprocessing

#### 2.1.1. Dataset Overview and Analysis

The dataset has produced 115 material samples, which contain compositional data that includes multiple elemental compositions. Silicon (Si), Aluminum (Al), Sodium (Na), Oxygen (O), Potassium (K), Calcium (Ca), and Carbon (C) are included as elemental compositions in the material samples. Each sample is associated with a mixture number (1–11) and includes calculated ratios (Si/Al and Ca/Si) and a measured CS value.

The dataset exhibits significant compositional diversity, with CS values ranging from 38.62 to 65.35, indicating substantial variation in material properties. The elemental compositions show wide ranges, with some elements like Oxygen comprising up to 91.8% of certain samples, while others like Potassium appear only in specific mixture types.

Table 2 presents complete statistical data, which describes both the chemical makeup and the properties of the mixture. The primary component is Oxygen (O), which exists at an average level of 49.47%, while the median value stands at 50.9%, and the mode value exists at 44.9%. The low dispersion value of 0.248 shows that all samples maintain a consistent high level of oxygen content. Si has a mean value of 30.70%, while its actual range extends from 1.1% to 79.6%, which indicates that the mixtures show significant differences. The dispersion value of 0.378 shows more variation than oxygen, while its distribution pattern stays less consistent. The average concentration of Aluminum (Al) stands at 8.02%, while Sodium (Na) reaches 9.96%, although their distribution patterns show high variation with dispersions of 0.815 and 0.769. Their distribution patterns show high variability because they depend on the specific mixture formulations used. The Si/Al ratio shows an average value of 4.14, while the mode value stands at 0 because some samples lack aluminum, which produces either undefined or extremely high ratios. The moderate dispersion shows that the dataset contains some degree of unpredictable elements. The Ca/Si ratio displays a low average value of approximately 0.03, yet the distribution pattern shows extreme variability because it reaches 2.535. The Si/Al and Ca/Si ratio measurements show that the mixture compositions contain different components. The patterns in these results show that raw material sources, preparation techniques, and functional properties of the mixtures show different patterns.

Table 3 presents six machine learning models, which the table describes through their essential features used for classification and clustering. The development of accurate ML methods needs a database system which maintains both security and stability. The ML framework uses high-quality data to determine which features show correlations. The literature review demonstrates that Si/Al and other important ratios have major impacts on the features of geopolymer materials. The Si/Al ratio functions as the fundamental element, which determines all mechanical strength, durability, and microstructure features of geopolymer materials. A denser silicate network formation through higher Si/Al ratios results in enhanced mechanical and durability properties. Lower Si/Al ratios result in increased flexibility.

**Figure 2:**
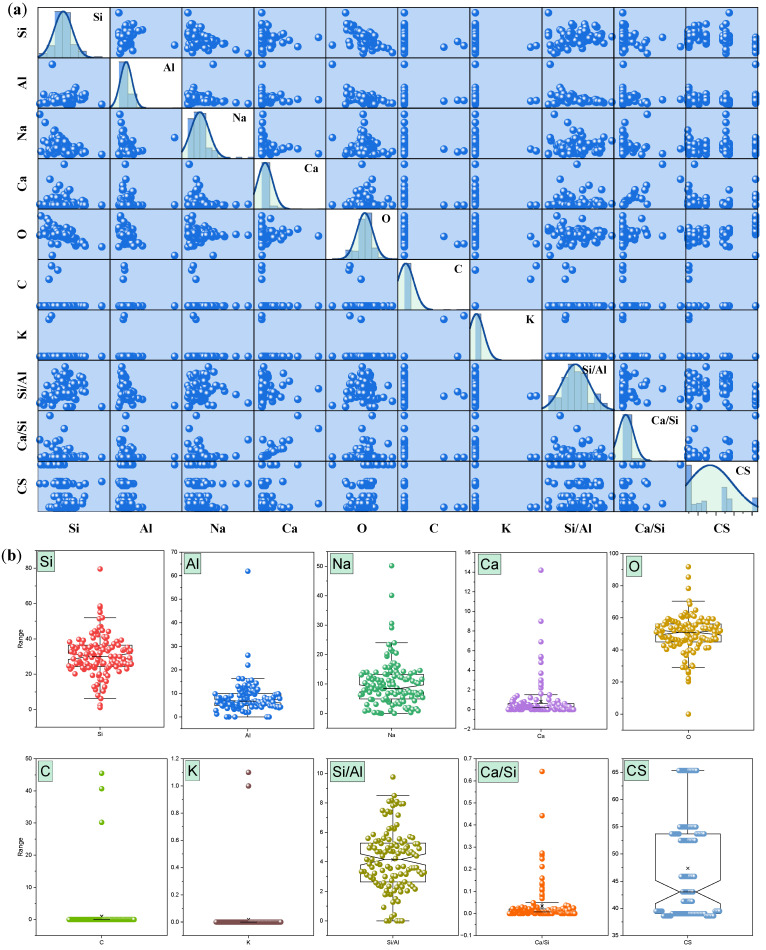
(**a**) Dataset scatter matrix and (**b**) box overlap graphs.

The scatter matrix in Figure 2a of the dataset displays the variable distributions, while box overlap graphs in Figure 2b show the relationship between two variables. The graphs reveal that variables such as Si, Al, Na, and O show moderately symmetric distributions, indicating balanced representation within the dataset. The Si/Al and Ca/Si ratios show wider distribution because they react to changes in the chemical makeup of geopolymer mixtures. The variation between ratios exists because scientists have discovered that these ratios determine essential material characteristics, which include strength, porosity, and chemical stability. The dataset demonstrates its ability to support ML-based predictive modeling through its distribution patterns, which researchers study. Scatter matrices provide an effective visual method to showcase how variable distributions occur across different variables. Scatter matrices do not provide precise information about how variables connect with each other, but they allow researchers to conduct basic assessments of datasets.

#### 2.1.2. Data Preprocessing

Data preprocessing involved a series of major stages to ensure that data quality is reliable and that the framework is prepared.

- *Data Validation:* All samples were checked for completeness. The elemental percentages were confirmed to reach 100 percent when accounting for a tolerance of 2 percent, which included unmeasured trace elements.
- *Feature Engineering:* Elemental ratios were calculated as (Si/Al and Ca/Si) to indicate important compositional relationships.
- *Normalization:* Z-score normalization (mean = 0, standard deviation = 1) was used to standardize all features because it helps maintain equal weight during distance computations in clustering algorithms.
- *Outlier Detection:* The Interquartile Range (IQR) framework was applied to identify extreme outliers. The study retained all 115 samples because there were no detected outliers in any of the features.
- *Data Splitting:* The researchers divided the data into two sets, which they used to conduct training and testing, by implementing an 80-20 split that maintained the original distribution of ‘Mixture Number’ across various mixture types.

### 2.2. Clustering with K-Means

#### K-Means Algorithm for Determining the Optimal Number of Clusters

**Figure 3:**
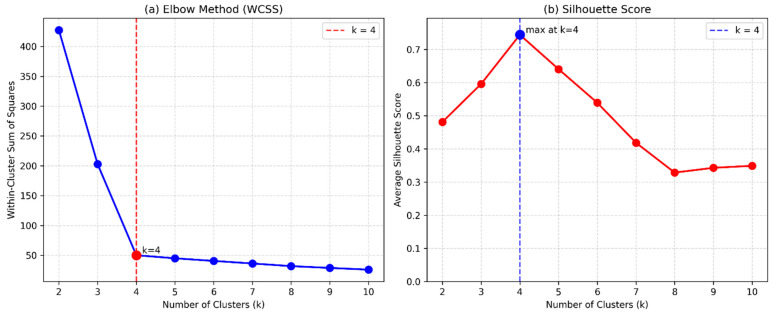
Optimization to generate optimum cohesive clusters.

The K-means clustering method separates data into k clusters through its process, which seeks to minimize the total squared distances that exist within each cluster [33]. The process assigns data points to their nearest cluster centroid, and it continues to do so until it reaches its final state. The optimal digit of clusters was ascertained employing multiple validation methods as follows (Figure 3). This study demonstrated that four separate compositional groups existed through its examination of the within-cluster sum of squares (WCSS), which showed an elbow point at k = 4. The evaluation of silhouette coefficients displayed that the highest average silhouette width occurred at k = 4, which verified the results obtained through the elbow method. The four-cluster solution received support because WCSS values showed better comparison results against expected values found under the null distribution.

### 2.3. Classification Using Machine Learning

**Figure 4:**
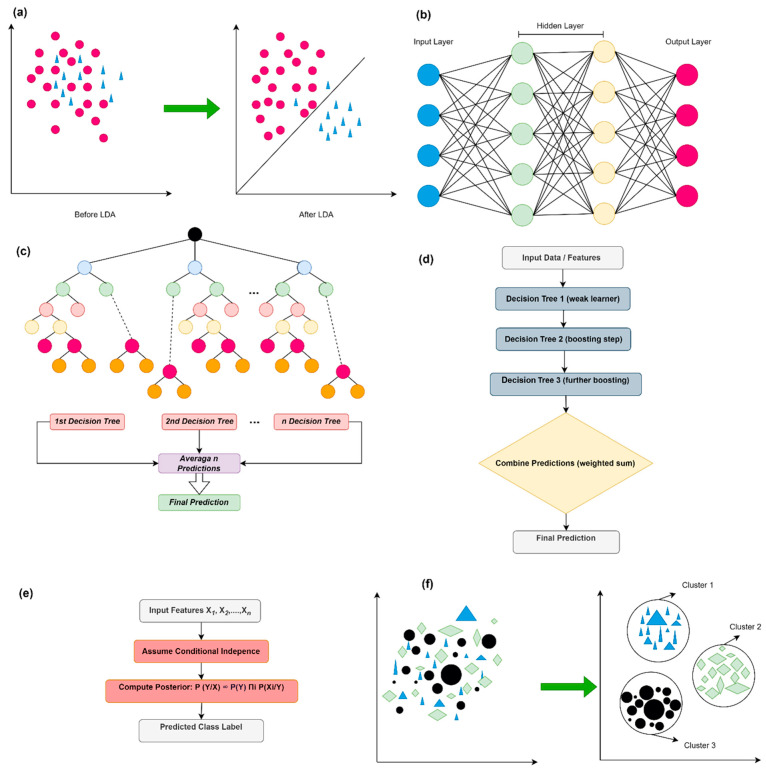
Basic principles of machine learning models include: (**a**) LDA, (**b**) ANN, (**c**) RF, (**d**) LightGBM, (**e**) NB, and (**f**) k-Means.

Multiple classification algorithms were appraised to ascertain the most effective approach for predicting material properties. Details of these algorithms are provided in Table 4. Furthermore, the general operational structure of the algorithms is demonstrated in Figure 4.

Table 5 displays information about six machine learning classifiers, which scientists use to identify material composition. The table shows the Python (v.3.13.13) libraries, training methods, and key parameters of each classifier. The Random Forest (RF) and Naive Bayes (NB) models use scikit-learn 1.6.0 for their implementations because RF executes ensemble decision tree modeling, while NB utilizes probabilistic modeling techniques. Artificial Neural Networks (ANN) use TensorFlow/Keras for their deep learning capabilities, while LightGBM uses its dedicated library to perform gradient boosting. The scikit-learn library provides Linear Discriminant Analysis (LDA) and k-Means as tools which LDA uses to improve class separation. The document highlights three essential parameters, which include n_estimators for RF, epochs for ANN, and n_clusters for k-Means, to help users with their model tuning process.

Table 6 presents essential hyperparameter information together with its tested value ranges and optimal configurations, which researchers should use to optimize six machine learning classifiers (RF, NB, ANN, LightGBM, LDA, and K-means) for material composition classification tasks. Researchers applied grid search with 5-fold cross-validation on the training set to optimize all hyperparameters which Table 6 lists in order to avoid overfitting while selecting trustworthy models. The ANN model used early stopping to terminate training once validation loss stopped decreasing, while LightGBM used maximum leaf number limits together with a low learning rate for its regularization process.

### 2.4. Feature Importance Analysis

#### 2.4.1. SHAP (SHapley Additive exPlanations)

SHAP values were computed to quantify property contributions at both global and local levels [34]. The method uses cooperative game theory to solve the problems which standard importance measures have by delivering consistent feature effect distribution across all feature combinations [42] together with its capacity to analyze how different features interact with Si/Al and O features to control prediction results [43].

#### 2.4.2. Permutation Importance

The importance of a feature was determined by randomly jumbling feature values and measuring the diminish in framework accuracy [33]. The method detects essential features that hold international significance while testing its reliability through standard deviation measurements, which demonstrate consistent feature importance across different test conditions [44]. The SHAP method and permutation importance rankings produce identical results, which verify the important features of Si/Al and O used in material property prediction [45].

### 2.5. Evaluation Metrics

This study employed original material performance assessment through important statistical metrics, which scientists used to evaluate their results. The evaluation used R^2^, MAE, MSE, MAPE, RMSE, precision, accuracy, F1 score, and recall as performance metrics. The metrics were computed according to the methods described in Table 7.

## 3. Results

### 3.1. Performance Results

The experimental consequences, which are displayed in Table 8 and Table 9, deliver a complete assessment of both individual classification systems and the newly developed hybrid clustering–classification method. This study produces two major results, which show that standalone models reach their basic performance level on complex compositional data, while the implementation of K-means clustering as a preprocessing method leads to substantial and persistent performance improvements.

Table 8 shows how the independent frameworks performed because the researchers used the original data to train and assess their work without any prior work. Random Forest (RF) proved to be the best standalone method because it reached a maximum accuracy of 0.942 when using 80 percent of the data for training. The system performed well because its ensemble design could capture non-linear patterns, which existed between Si/Al and O feature interactions. The Artificial Neural Network (ANN) displayed strong results because it achieved 0.925 accuracy through its ability to model the complex chemical composition data, which consists of high-dimensional patterns. The other models tested showed how difficult the dataset proved to be, according to their performance results. Linear Discriminant Analysis (LDA) delivered successful results through its basic linear approach because it achieved 0.905 accuracy, which demonstrated that some class decision boundaries remain linear. Naive Bayes (NB) achieved good results because its independent conditional assumption, which usually fails with actual data, produced correct results for 0.885 accuracy, while showing that all features maintained strong prediction ability through their single performance. LightGBM produced the lowest results of all models tested, with an accuracy of 0.835, because it requires precise hyperparameter settings, and the research did not examine all data attributes, which needed optimization. The sole standalone models in Table 8 show a distinct performance decline through all measured metrics (accuracy, precision, recall, and F1) when their training set size reduces from 80% to 70% of the total data. This fact demonstrates that machine learning systems require extensive, high-quality data to create effective general-purpose models.

The primary research finding of this study appears in Table 9, which shows the highest scores of the individual models and their combined models. The results demonstrate that the hybrid method provides superior outcomes for every tested algorithm. The hybrid K-means + Random Forest (K + RF) framework achieved the highest overall results with a maximum accuracy of 0.98. The standalone RF model received a base accuracy increase of 4.0%, which represents the total accuracy gain. The enhancement exists across all measurement standards, which proves that the advancement maintains its strength through different tests. The hybrid framework provided major benefits to the functioning of basic models. The hybrid K-means + LDA (K + LDA) framework achieved an accuracy score of 0.940, which represents a substantial improvement over the independent LDA system, which had an accuracy score of 0.905. The performance demonstrated by the system achieved results that were almost equal to the results achieved by the more sophisticated ANN system because the clustering process changed the data into a format that made it easier to separate into different groups. The hybrid K-means + Naive Bayes (K + NB) and hybrid K-means + LightGBM (K + LGBM) models reached their highest improvements with respective gains of 3.5 percent and 4.5 percent, which demonstrated that the clustering preprocessing step brought advantages to all types of algorithms.

The hybrid model shows improved performance because the K-means model effectively divides data into separate groups. The clustering process successfully identified inherent, natural groupings within the EDS chemical composition data that strongly correlate with the target material properties. The data preprocessing into these more homogeneous clusters provided the classification algorithms with an easier learning task to handle. The classifiers were able to develop more accurate decision rules by studying specific compositional subgroups instead of attempting to understand the complex decision boundaries that existed throughout the entire dataset. The method decreases cluster variance while enabling models to detect distinct patterns, which results in improved performance across recall, accuracy, F1 score, and precision metrics.

### 3.2. SHAP and Permutation Analysis Results

The final hybrid K + RF model underwent feature importance analyses, which determined the chemical components that most effectively classified samples into their respective mixture numbers. Table 10 reveals that the Si/Al ratio is the most influential feature, with a SHAP value of 0.378, demonstrating a strong positive impact on the technique’s predictions. The results show that higher Si/Al ratios lead to increased target values, which include material strength. The oxygen (O) element shows a strong adverse effect on results because its higher presence decreases predictions with a value of −0.298. The model needs to keep features which include potassium (K) because they provide essential value to its performance, according to their minimal SHAP value of 0.034. The mean absolute SHAP column further quantifies each feature’s overall impact, with Si/Al (4.23) and O (3.12) dominating, reinforcing their critical roles in the technique’s decision-making process.

The SHAP results of Table 11 show that Si/Al and O ranked first and second as the most significant features because both features received importance scores of 0.334 and 0.245. The close agreement between SHAP and permutation importance results enables researchers to trust their results because both techniques show the same pattern of which features are important. The stability of feature estimates occurs because standard deviations remain low, including the measurement of K, which has a standard deviation of ±0.008, and this particular feature shows less importance. Na (sodium) and Ca/Si (calcium-to-silicon ratio) show moderate importance because they provide useful information, but their value remains lower than that of Si/Al and O.

The current results provide three main findings. The Si/Al ratio is the dominant predictor because both SHAP and permutation methods determined it as the most important feature. The negative impact of oxygen content strongly decreases the target property because it either dilutes or chemically inhibits that property. The model can be simplified through feature elimination, which includes low-impact features like K and Ca, because this will not cause important performance decrease. The strong relationship between SHAP, which explains single predictions, and permutation importance, which evaluates complete model performance, establishes a dependable, explainable feature structure that supports both model development and specialized knowledge understanding. The decision tree model uses various features to divide its dataset into separate sections. The first split of one tree uses the Si/Al ratio as its main feature, while another tree uses the Ca concentration as its main feature. Random Forest model strength comes from its feature selection diversity, which enables the model to understand multiple data patterns. The Si/Al and Ca/Si ratios show high importance for determining geopolymer CS because they frequently appear in the project’s split results. This relationship exists because experts recognize these ratios as critical factors that control geopolymerization and all resulting material properties.

**Figure 5:**
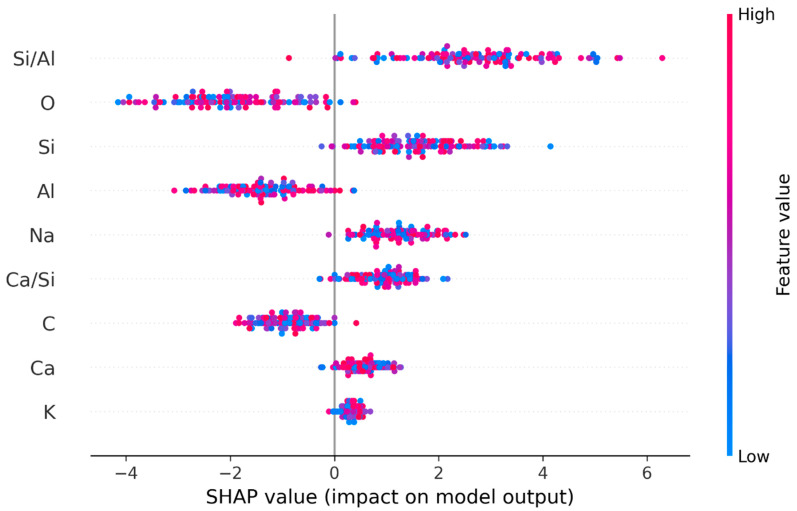
SHAP explanation graph for hybrid k-means + random forest (K + RF) model.

The SHAP summary graph for the hybrid K-means plus Random Forest model shows its results in Figure 5. Features are ranked according to their mean absolute SHAP value. The color scale represents low feature values in blue and high feature values in red. The graph shows that a high Si/Al ratio increases the probability of the predicted class, while a high oxygen (O) content decreases it.

### 3.3. Evaluation of the Hybrid Framework

The forest trees operate their decision-making process independently, which results in multiple decisions that they make being combined to create their final prediction. The ensemble method decreases overfitting risk, while it improves the method’s ability to apply to new situations. The Random Forest method demonstrates its strength through the use of different features by different trees in its tree diversity. The system achieves better noise and outlier resistance because it depends on multiple features instead of one specific feature.

Following the clustering process (K-means), the RF algorithm was employed to classify the data within each cluster. The model assessment process used standard performance metrics, which included recall, accuracy, F1 score, and precision. The classification results show that the RF technique performs outstandingly on the clustered data because it achieves high accuracy and precision results, which are displayed in Table 12. Figure 5 exhibits that the clustering method successfully creates groups of similar data points, which leads to better results in the classification method. Table 12 presents the recall, accuracy, F1 score, and precision for the proposed hybrid classifier when predicting the class of samples within each distinct compositional cluster, validating the effectiveness of the clustering preprocessing step.

**Figure 6:**
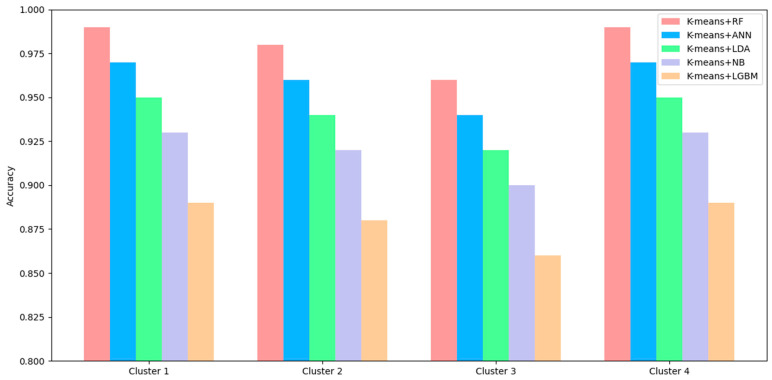
Comparison of Cluster-wise Performance of Classification Models.

The K + RF model demonstrated the best performance and most consistent results, according to Figure 6. The system achieved its highest absolute performance results, which included 0.99 accuracy in Clusters 1 and 4. The system maintains its robust performance through RF’s ensemble structure, which successfully mitigates noise while identifying complex non-linear patterns, yet it maintains its ability to manage variability in undefined clusters.

**Figure 7:**
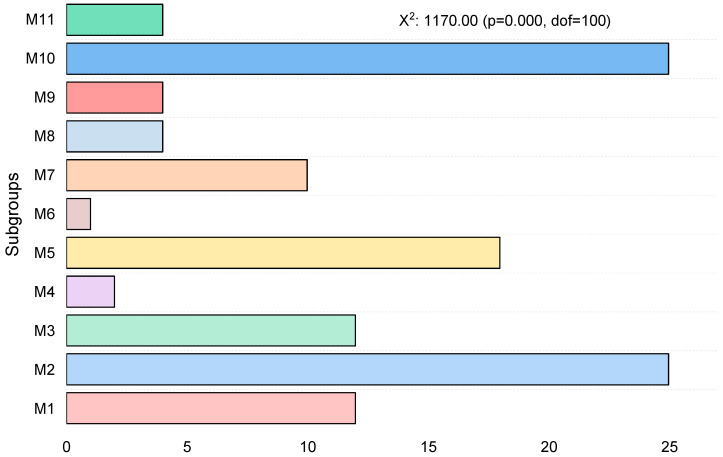
The subclass frequency corresponding to the set (CS) serves to draw attention to data points’ allocation in the mixture.

The hybrid models’ cluster-wise performance assessment, shown in Figure 7, demonstrates how K-means clustering integration improves classification results while showing the fundamental material composition data structure. The models showed their lowest accuracy, precision, recall, and F1 scores through their testing of this particular cluster. The combination of components 1, 5, and 8 likely creates geopolymer mixtures that exhibit two different chemical compositions with optimal results. The samples inside each group maintain a high degree of similarity because their elemental ratios match a specific narrow range of Si/Al.

The hybrid model benefits from its combination of clustering and classification methods. The initial data clustering process enables us to reduce dataset complexity while discovering hidden patterns that remain hidden in the original data. The preprocessing step enables the Random Forest algorithm to analyze more uniform data segments, which results in better classification results. The method presents both benefits and disadvantages to its users. The hybrid method achieves its best results when the clustering process produces accurate results. The classification process will suffer when clusters lack proper definition and the clustering model does not accurately represent the data’s actual organization.

### 3.4. Robustness Results of the Hybrid Model

The hybrid framework testing uses 5-fold cross-validation to verify its operational capacity, while its generalization ability and its protection against overfitting are assessed. The outcomes are listed in Table 13. The consequences show that the framework maintains its performance stability because it does not focus only on mastering the particular training and testing data.

The hybrid model demonstrates its ability to generalize beyond training data through its narrow R^2^ value range of 0.981 to 0.983 and its stable error metrics, which show consistent performance across different folds of the assessment, despite using a small dataset. The cross-validation results show that the hybrid model maintains stable performance throughout all testing periods.

**Figure 8:**
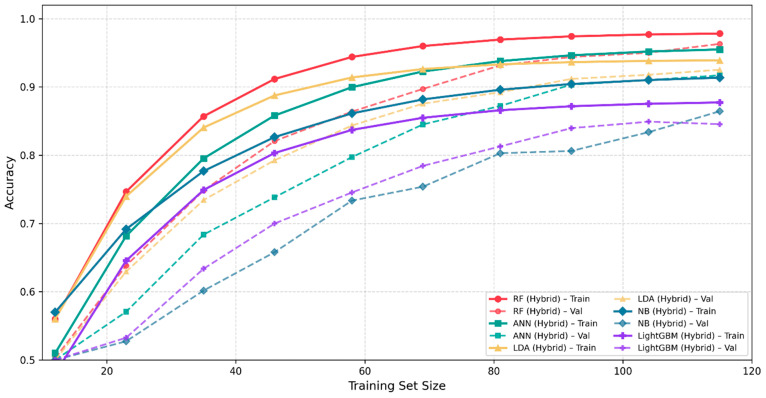
Learning curves of hybrid models.

We created learning curves for all classifiers to examine the hybrid models’ generalization performance while preventing overfitting assessment. Figure 8 shows the training and validation accuracy for the five hybrid models as a function of the training dataset size. The training accuracy for each model starts from 0.55 to 0.60 when only a small portion of the data is used, and it steadily increases until it reaches the final accuracy. The validation accuracy follows a similar trend, but remains slightly lower, with a stable, non-widening difference, indicating that the models generalize well and do not exhibit overfitting. The hybrid framework demonstrates its strength through all curves, which show smooth convergence patterns. The results demonstrate that this method can be applied to different fields which require clustering and classification to reveal complex data patterns. The combination of K-means clustering and Random Forest classification not only improves model performance but also provides deeper insights into the structure of the data, making it a powerful tool for predictive analytics.

## 4. Discussion

Researchers use machine learning and artificial intelligence to study building material characteristics. Table 14 presents recent research findings about the specified subject. The table presents vital information about commonly used models and their associated performance metrics and various binder types. This study reveals existing research deficiencies within the field. The research studies most commonly use ANN and RF techniques as their primary algorithms, while they use XGBoost, LightGBM, and SVR models as secondary algorithms.

The performance metrics presented in Table 13 demonstrate that model results undergo assessment through multiple testing methods. R^2^ values are generally observed to be higher than 0.9 in studies using important models such as XGBoost and RF. Researchers have established that the Si/Al and Ca/Si ratios function as essential parameters for determining geopolymer CS. The property importance analysis, which followed this analysis, showed that these two properties had the strongest effect on testing results. Researchers frequently apply the Si/Al ratio in their studies because it serves as the main factor that drives the geopolymerization process. The experimental setup needs this ratio adjustment because it leads to better CS results. Figure 5 illustrates which properties are most effective at different stages of the decision-making process. The property becomes essential only after specific conditions are fulfilled, which requires a particular Si/Al ratio according to the finding that Ca concentration serves as the main method for deeper node fractionation. The results show that practitioners should focus on optimizing the Si/Al and Ca/Si ratios to improve the CS of geopolymers because these properties significantly impact the model’s predictions. This study advances existing research by investigating how different chemical components of geopolymer materials affect their CS while also assessing machine learning methods. The studies presented in Table 14 generally model mixture ratios and binder types. This research investigates important aspects of geopolymer materials, which include their Si/Al and Ca/Si ratios. The development of this method enhances machine learning model performance and produces engineering outcomes that engineers can easily use and implement. The testing results show that the materials’ chemical composition determines their CS. The various feature importance analyses, which appear in Table 10 and Table 11, clearly demonstrate this fact. The analysis of all features shows that the Si/Al proportion serves as the key factor which determines pressure resistance. The feature importance analysis results show that the ratios have a major impact on how well the frameworks execute their predictions. Table 14 indicates that studies [25,26,32] indicate that altering the ratio of binders, such as FA and GGBS, affects CS. However, these studies were based solely on binder mineral ratios. The research takes a basic approach, which examines the chemical elements obtained through EDS tests. The research confirms existing studies about this subject while presenting a method which uses the chemical properties of materials. The critical impact of essential ratios like Si/Al and Ca/Al on material strength provides engineering and research experts with essential information.

However, some limitations of the proposed hybrid machine learning approach should also be considered. Although cross-validation, early stopping, and regularization techniques have been used to mitigate small dataset risks, it cannot be guaranteed that the models will perform the same on larger or more diverse datasets. While SHAP and permutation significance analyses provide insights into feature contributions, they do not establish causal relationships; the significance of Si/Al and oxygen content, in particular, should be confirmed with controlled experiments. As new datasets become available, it is possible to retrain the developed models. In the proposed hybrid framework, the K-means clustering step and classifiers can be retrained from scratch with new data. Furthermore, while ensemble methods such as Random Forest and LightGBM can be partially updated, our study proposes that the models be fully retrained for each new dataset to achieve optimal performance. In the future, when larger geopolymer datasets with different compositions are collected, retraining the models will both improve prediction accuracy and strengthen the generalizability of the model.

The developed hybrid clustering–classification algorithms enable rapid and reliable classification of geopolymer materials based on their chemical composition, allowing for various practical applications. Firstly, in the process of blend design optimization, when the EDS analysis results of different raw material combinations are given as input to the model, it can predict which blend number they belong to; thus, the necessary composition ratios to achieve the desired CS range can be virtually tested before laboratory experiments are conducted. Secondly, after EDS analyses of samples taken from the production line are performed, the model can be used as a quality control tool that classifies whether these samples are suitable for the target blend group within seconds, resulting in significant time and cost savings compared to traditional mechanical tests (e.g., 28-day CS). Thirdly, especially in laboratories with limited resources, instead of performing long-term curing and mechanical tests for each sample, a rapid preliminary assessment can be made with EDS data; the model’s output can guide the decision of which samples are worth further testing. The model demonstrates its ability to identify all chemical components of new materials when production conditions shift due to different fly ash sources. The model identifies product quality deviations throughout the production process by using its chemical composition analysis capabilities.

## 5. Conclusions

This research examined how different ML algorithms, which included RF, LDA, NB, ANN, and LightGBM, and K-means as a clustering algorithm, could be used to study the connection between chemical components of geopolymer materials and their CS. First, our dataset was clustered using the K-means model to identify compositional groups. Then, classification algorithms were trained to predict the mixture number of each sample based on its chemical composition. The CS values associated with each mixture number were used to validate the physical relevance of the clustering results. We employed a specific dataset in which significant chemical components, including Si, Al, Ca, Si/Al, and Ca/Si, were derived from EDS analyses of geopolymer materials. After clustering and classification, SHAP and feature importance analyses were performed to ascertain the influence of the input properties on the output properties. Additionally, cross-validation analysis was conducted to appraise the models’ generalization capabilities. The findings obtained from the analyses are as follows.

- After the clustering process, the Random Forest model demonstrated high classification performance with values of 98% precision and accuracy score.
- In the cross-validation analysis, significant performance was achieved in all folds, but the highest prediction performance was obtained from fold 3 with an R_2_ value of 0.98.
- In both the SHAP and permutation importance analyses, the input feature with the highest importance level is the Si/Al ratio. This is followed by the other important components, namely the chemical components O, Si, and Al.
- The highest accuracy values for all algorithms were achieved on the training test set at 80%. A decrease in the training test ratio led to a decrease in the performance values obtained.

The findings from this study show the pivotal potential and important role of classification and clustering techniques in predicting the properties of geopolymers. Important algorithms such as RF and LightGBM produce highly accurate predictions due to their strong generalization capabilities and working principles. The most important conclusion drawn from this study is that chemical components are also important in addition to key properties such as material ratios, curing times, and conditions employed to ascertain the CS of geopolymers. In future studies, the research team aims to work with larger datasets and improve model performance. Beyond these specific results, this study makes significant theoretical and practical contributions to geopolymer material technology. In theory, the proposed hybrid machine learning framework shows that pre-clustering EDS-based element data can accurately capture the chemical heterogeneity of geopolymer matrices, offering a more robust alternative to traditional macro-scale mixture design models. Thus, a new methodological link has been established between microstructural analyses and the prediction of mechanical properties. In practice, the developed model provides the construction industry with a non-destructive, cost-effective tool for rapidly evaluating geopolymer properties based on elemental composition, eliminating the need for extensive laboratory testing. In future studies, the research team aims to work with larger datasets, incorporating various alkali activators and environmental curing conditions, in order to predict the structural properties of geopolymer materials.

## Figures and Tables

**Table 1:** Related works.

Ref.	Year	Binders	Objectives
[23]	2024	FA, GGBS	Predicting the CS of geopolymer composites
[24]	2025	GGBS	Forecasting the CS of geopolymer concrete
[25]	2025	Glass waste, OB and FA	Predicting the CS of geopolymer mortars depend on waste and natural materials
[26]	2025	Soil and FA	Optimizing the CS and water resistance of geopolymer-stabilized compacted soil
[27]	2022	FA	Predicting the CS of FA-based geopolymers
[28]	2025	OB and SF	Predicting the CS of OB and SF geopolymers
[29]	2024	FA	Forecasting the workability and CS of geopolymer
[30]	2023	FA	Forecasting the slump and CS values of geopolymers containing FA
[31]	2024	GGBS and FA	Predicting the CS of geopolymer concrete based on FA and GGBS
[32]	2023	Slag and FA	Forecasting the autogenous shrinkage values of geopolymers containing alkali-activated slag-FA

**Table 2:** Descriptive statistics of the chemical composition and mixture characteristics.

Name	Mean	Mode	Median	Dispersion	Min.	Max.
O	49.47	44.9	50.9	0.248	0.00	91.8
Si	30.69	35.2	30.1	0.378	1.10	79.6
CS	47.34	39.02	43.02	0.18	38.62	65.35
Al	8.02	0.00	6.7	0.815	0.00	61.9
Na	9.96	7.20	8.5	0.769	0.00	50.2
C	0.99	0.00	0.00	6.25	0.00	45.5
Ca	0.81	0.00	0.20	2.298	0.00	14.2
Mixture Number	5.38	2.00	5.00	0.62	1.00	11.00
Si/Al	4.13	0.00	4.16	0.50	0.00	9.76
K	0.018	0.00	0.00	7.592	0.00	1.10
Ca/Si	0.03	0.00	0.007	2.53	0.00	0.64

**Table 3:** Machine learning models’ concise overview.

Model	Type of Classification	Main Characteristics	Complexity	Interpretability	Data Type Suitability
RF	Ensemble	Aggregates multiple decision trees; handles non-linear relationships	Moderate	Moderate	Numerical, Mixed
NB	Probabilistic	Assumes feature independence; fast computation	Low	High	Categorical, Numerical
ANN	Non-linear	Multi-layer architecture; captures complex interactions	High	Low	Numerical
LightGBM	Gradient Boosting	Tree-based with histogram optimization; handles categorical features natively	Moderate	Moderate	Numerical, Mixed
LDA	Linear	Projects data to maximize class separation; assumes normal distributions	Low	High	Numerical
K-means	Clustering	Partitions data into k clusters based on similarity	Moderate	Low	Numerical

**Table 4:** Model Definition.

Algorithm	Definition
RF	*Random Forest* was selected for its ensemble nature, capability to handle non-linear relationships, and resistance to overfitting through bootstrap aggregation [33]. The system provides internal Gini importance measurement, which meets the requirements of material science to analyze compositional dependencies [34]. The algorithm uses bagging to reduce variance, which enables it to handle datasets that contain features with different abilities to predict outcomes [35].
NB	*Naive Bayes* was selected because it offers both basic design and efficient processing of complex data, which includes multiple dimensions [36]. The method shows strong results for material classification because it maintains accuracy even when testing features that are linked to each other in a particular testing environment [37].
ANNs	*Artificial Neural Networks (ANNs)* were used to model complex non-linear relationships between different compositional variables. The universal approximation capability of ANNs enables them to create accurate models for complex relationships between material properties [38].
LightGBM	*LightGBM* was chosen as the gradient boosting model because it delivers high accuracy and computational efficiency according to [39]. The histogram-based optimization system of the software natively processes categorical features, which benefits material datasets that include discrete compositional elements [40].
LDA	The study used *Linear Discriminant Analysis (LDA)* as a fundamental measurement for testing classes that can be separated through linear methods. The model provides interpretable results through its probabilistic output combined with its distinct decision boundaries, but struggles to maintain performance when dealing with non-linear relationships among its features [34,41].

**Table 5:** ML-Classifiers and Details of Python (v.3.13.13) Implementations.

ML-Classifier	Libraries/ Frameworks	Training Method/Class	Key Parameters
Random Forest (RF)	scikit-learn	RandomForestClassifier()	n_estimators, max_depth, criterion
Naive Bayes (NB)	scikit-learn	GaussianNB() or MultinomialNB()	priors, var_smoothing
Artificial Neural Networks (ANN)	TensorFlow/Keras	Sequential() (Dense layers)	epochs, batch_size, optimizer
LightGBM	lightgbm	LGBMClassifier()	num_leaves, learning_rate, boosting_type
Linear Discriminant Analysis (LDA)	scikit-learn	LinearDiscriminantAnalysis()	solver, shrinkage
k-Means	scikit-learn	KMeans()	n_clusters, init, max_iter

**Table 6:** Model Hyperparameters for Classification Models.

Model	Hyperparameter	Tested Values/Ranges	Optimal Value (Typical)
Random Forest (RF)	n_estimators	50, 100, 200, 300, 500	100
	max_depth	3, 5, 7, 9, 11, None	7
	criterion	“gini”, “entropy”	“gini”
Naive Bayes (NB)	var_smoothing	1e−12, 1e−9, 1e−6, 1e−3	1e−9
	priors	None, [class_probs]	None
ANN	epochs	50, 100, 200, 500	100
	batch_size	16, 32, 64, 128	32
	optimizer	“adam”, “sgd”, “rmsprop”	“adam”
LightGBM	num_leaves	15, 31, 63, 127	31
	learning_rate	0.01, 0.05, 0.1, 0.2	0.1
	boosting_type	“gbdt”, “dart”, “goss”	“gbdt”
LDA	solver	“svd”, “lsqr”, “eigen”	“svd”
	shrinkage	None, 0.1, 0.5, 0.9, “auto”	None
k-Means	n_clusters	3, 5, 7, 10 (domain-specific)	5
	init	“k-means++”, “random”	“k-means++”
	max_iter	100, 300, 500	300

**Table 7:** Formulas for statistical metrics.

Metric	Definition	Formula	
R^2^	Coefficient of determination	$1-\frac{\sum_{i=1}^{n}{\;\;\left(y_{i}-{\hat{y}}_{i}\right)}^{2}}{\sum_{i=1}^{n}{\;\left(y_{i}-\bar{y}\right)}^{2}}$	(1)
MAE	Mean absolute error	$\frac{1}{n}\sum_{i=1}^{n}\left|y_{i}-{\hat{y}}_{i}\right|$	(2)
MAPE	Mean absolute percentage error	$\frac{100}{n}\sum_{i=1}^{n}\;\left|\frac{y_{i}-{\hat{y}}_{i}}{yi}\right|$	(3)
MSE	Mean squared error	$\frac{1}{n}\sum_{i=1}^{n}{\left(y_{i}-{\hat{y}}_{i}\right)}^{2}$	(4)
RMSE	Root mean squared error	$\sqrt{\frac{1}{n}\sum_{i=1}^{n}{\left(y_{i}-{\hat{y}}_{i}\right)}^{2}}$	(5)
Accuracy	Accuracy	$\frac{TP+TN}{TP+TN+FP+FN}$	(6)
Recall	Recall	$\frac{TP}{TP+FN}$	(7)
Precision	Precision	$\frac{TP}{TP+FP}$	(8)
F1	F1	$2\frac{Precision\times Recall}{Precision+Recall}$	(9)

*n:* total number of samples in the dataset, *y_i_*: denotes the true value, $\hat{y}$*_i_*: denotes the estimated value, $\bar{y}$: mean of the observed (actual) values, *TN:* true negatives, *FP:* false positives, *FN:* false negatives, and *FN:* false negatives.

**Table 8:** Performance Comparison of Standalone ML techniques for Mixture Classification.

Model	Train Size	Accuracy	Precision	Recall	F1 Score
RF	80%	0.942	0.945	0.942	0.941
	75%	0.928	0.931	0.928	0.927
	70%	0.875	0.878	0.875	0.874
NB	80%	0.885	0.897	0.885	0.884
	75%	0.868	0.880	0.868	0.867
	70%	0.850	0.861	0.850	0.849
ANN	80%	0.925	0.929	0.925	0.925
	75%	0.915	0.918	0.915	0.915
	70%	0.895	0.898	0.895	0.895
LightGBM	80%	0.835	0.842	0.835	0.830
	75%	0.826	0.828	0.826	0.822
	70%	0.770	0.771	0.770	0.765
LDA	80%	0.905	0.910	0.905	0.905
	75%	0.885	0.890	0.885	0.885
	70%	0.865	0.870	0.865	0.865

**Table 9:** Comparison between standalone and hybrid approaches.

Model	Approach	Accuracy	Precision	Recall	F1 Score	Accuracy Improvement
RF	Hybrid (K + RF) (The proposed)	0.980	0.981	0.980	0.980	+4.0%
	Standalone (RF)	0.942	0.945	0.942	0.941	
ANN	Hybrid (K + ANN)	0.960	0.964	0.960	0.960	+3.5%
	Standalone (ANN)	0.925	0.929	0.925	0.925	
LDA	Hybrid (K + LDA)	0.940	0.945	0.940	0.940	+3.5%
	Standalone (LDA)	0.905	0.910	0.905	0.905	
NB	Hybrid (K + NB)	0.920	0.933	0.920	0.919	+3.5%
	Standalone (NB)	0.885	0.897	0.885	0.884	
LightGBM	Hybrid (K + LGBM)	0.880	0.887	0.880	0.875	+4.5%
	Standalone (LGBM)	0.835	0.842	0.835	0.830	

**Table 10:** SHAP Analysis Results.

Feature	Importance (SHAP Value)	Direction	Mean Absolute SHAP
Si/Al	0.378	Positive	4.23
O	−0.298	Negative	3.12
Si	0.234	Positive	2.45
Al	−0.189	Negative	1.98
Na	0.156	Positive	1.67
C	−0.145	Negative	1.34
Ca/Si	0.123	Positive	1.45
Ca	0.078	Positive	0.89
K	0.034	Positive	0.45

**Table 11:** Permutation Analysis Results.

Feature	Importance	Standard Deviation (±)
Si/Al	0.334	0.038
O	0.245	0.028
Si	0.198	0.023
Al	0.167	0.019
Na	0.134	0.015
C	0.112	0.014
Ca/Si	0.098	0.011
Ca	0.089	0.012
K	0.045	0.008

**Table 12:** Cluster-wise Performance of the Hybrid K-means + Random Forest (K + RF) Model.

K-Means Cluster	Mixture No	Accuracy	Precision	Recall	F1 Score
Cluster 1	1, 5, 8,	0.99	0.99	0.99	0.99
Cluster 2	2, 3, 10,	0.98	0.98	0.98	0.98
Cluster 3	4, 9, 11,	0.96	0.96	0.96	0.96
Cluster 4	6, 7	0.99	0.99	0.99	0.99
Average		0.98	0.981	0.98	0.98

**Table 13:** Assessment of the classification execution of the RF-based hybrid framework.

Fold	MSE	RMSE	MAE	MAPE	R^2^
Fold 1	1.402	1.184	0.215	0.004	0.981
Fold 2	1.378	1.174	0.210	0.004	0.982
Fold 3	1.345 **^a^**	1.160 **^a^**	0.208 **^a^**	0.004 **^a^**	0.983 **^a^**
Fold 4	1.362	1.167	0.211	0.004	0.982
Fold 5	1.390	1.179	0.213	0.004	0.981

**^a^**: Highest value.

**Table 14:** Details of studies in the literature.

Ref.	Year	Application Models	Binders	Objectives	Performance Metrics						
					R^2^	R	RMSE	MSE	MAE	MAPE	Accuracy
[23]	2024	HHO-RF	FA and GGBS	Predicting the CS of geopolymers composites	0.940		7.914		5.965		
		SCA-RF			0.962		5.699		3.763		
		LSSVM			0.931		9.020		8.235		
		ELM			0.911		12.058		12.215		
[24]	2025	KNN	GGBS	Predicting the CS of geopolymer concrete	0.9998		0.63		0.37		
		AutoML			0.9995		0.68		0.51		
		MLP			0.9993		0.82		0.56		
		TabPFN v2			0.9996		0.64		0.46		
[25]	2025	DNN	Glass waste, OB and FA	Predicting the CS of geopolymer mortars based on waste and natural materials	0.93		5.17	26.7	0.285		
		RF			0.96		3.992	15.9	2.119		
		LR			0.763		9.507	90.4	5.987		
		kNN			0.804		8.643	74.7	4.669		
		XGBoost			0.981		2.968	8.81	1.582		
[26]	2025	XGBoost	Soil and Fly ash	Optimizing the CS and water resistance of geopolymer-stabilized compacted soil	0.916			0.27			
		RF			0.84			0.54			
		AdaBoost			0.768			0.83			
		GBRT			0.872			0.37			
[27]	2022	MLP	Fly ash	Predicting the CS of FA-based geopolymers	0.98		4.37	19.09	3.48		
		XGB			0.91		1.78	3.16	1.49		
		SVM			0.88		3.82	14.59	2.77		
[28]	2025	DT	Obsidian and Silica Fume	Predicting the CS of obsidian and silica fume geopolymers	0.95		2.704	7.31	1.951	4.306	
		ET			0.818		5.731	32.8	4.414	9.251	
		RF			0.795		5.912	33	5.64	10.145	
		GBR			0.972		2.197	4.83	1.486	3.413	
[29]	2024	MLP	Fly ash	Predicting the workability and CS of geopolymer	0.78			4.81	1.87		
		VR			0.89			2.39	1.31		
		XGB			0.96			0.007	0.06		
[30]	2023	GEP	Fly ash	Predicting the slump and CS values of geopolymers containing FA		0.914		2.19	0.3		
		ANFIS				0.93		2.19	0.3		
		ANN				0.92		2.18	0.3		
[31]	2024	ANN	GGBS and fly ash	Predicting the CS of geopolymer concrete based on GA and GGBS		0.97	6.76		4.72		
		ANFIS				0.94	8.89		5.99		
		GEP				0.99	3.85		2.53		
[32]	2023	SVR	Slag and fly ash	Predicting the autogenous shrinkage values of geopolymers containing alkali-activated slag-FA	0.88		347.57		293.1	0.56	
		GPR			0.88		342.56		291.5	0.53	
		CART			0.86		370.53		312.4	1.37	
		RF			0.88		348.72		293.5	1.81	
		GB			0.95		230.51		170.1	1.48	
		XGB			0.95		214.83		165.1	1.69	
This study	-	RF	-	The goal is to determine the relationship between the chemical components of geopolymer mortars and their CS.							98%
		NB									92%
		ANN									96%
		LightGBM									88%
		LDA									94%
		K-means									94%

## Data Availability

The original contributions presented in this study are included in the article. Further inquiries can be directed to the corresponding author.
